# Perioperative Cerebral Microbleeds After Adult Cardiac Surgery

**DOI:** 10.1161/STROKEAHA.118.023355

**Published:** 2018-12-21

**Authors:** Nikil Patel, Caroline Banahan, Justyna Janus, Mark A. Horsfield, Anthony Cox, Xingfeng Li, Laurie Cappellugola, Jordan Colman, Vincent Egan, Peter Garrard, Emma M.L. Chung

**Affiliations:** 1 From the Department of Cardiovascular Sciences, University of Leicester, United Kingdom (N.P., J.J., M.A.H., L.C., E.M.L.C.); 2 NIHR Leicester Biomedical Research Centre–Cardiovascular Theme, Glenfield Hospital, United Kingdom (N.P., E.M.L.C.); 3 Department of Medical Physics, University Hospitals of Leicester NHS Trust, United Kingdom (C.B., E.M.L.C.); 4 Neuroradiology Department, St George’s Hospital, London, United Kingdom (A.C.); 5 Department of Clinical Neurosciences, St George’s, University of London, United Kingdom (N.P., X.L., J.C., P.G.); 6 Department of Psychiatry and Applied Psychology, University of Nottingham, United Kingdom (V.E.).

**Keywords:** cardiac surgery, cardiopulmonary bypass, cerebellum, cognition, hemorrhage, magnetic resonance imaging

## Abstract

**Background and Purpose—:**

Cerebral microbleeds (CMBs) have been observed using magnetic resonance imaging in patients with cardiovascular risk factors, cognitive deterioration, small vessel disease, and dementia. They are a well-known consequence of cerebral amyloid angiopathy, chronic hypertension, and diffuse axonal injury, among other causes. However, the frequency and location of new CMBs postadult cardiac surgery, in association with cognition and perioperative risk factors, have yet to be studied.

**Methods—:**

Pre- and postsurgery magnetic resonance susceptibility-weighted images and neuropsychological tests were analyzed from a total of 75 patients undergoing cardiac surgery (70 men; mean age, 63±10 years). CMBs were identified by a neuroradiologist blinded to clinical details who independently assessed the presence and location of CMBs using standardized criteria.

**Results—:**

New CMBs were identified in 76% of patients after cardiac surgery. The majority of new CMBs were located in the frontal lobe (46%) followed by the parietal lobe (15%), cerebellum (13%), occipital lobe (12%), and temporal lobe (8%). Patients with new CMBs typically began with a higher prevalence of preexisting CMBs (*P*=0.02). New CMBs were associated with longer cardiopulmonary bypass times (*P*=0.003), and there was a borderline association with lower percentage hematocrit (*P*=0.04). Logistic regression analysis suggested a ≈2% increase in the odds of acquiring new CMBs during cardiac surgery for every minute of bypass time (odds ratio, 1.02; 95% CI, 1.00–1.05; *P*=0.04). Postoperative neuropsychological decline was observed in 44% of patients and seemed to be unrelated to new CMBs.

**Conclusions—:**

New CMBs identified using susceptibility-weighted images were found in 76% of patients who underwent cardiac surgery. CMBs were globally distributed with the highest numbers in the frontal and parietal lobes. Our regression analysis indicated that length of cardiopulmonary bypass time and lowered hematocrit may be significant predictors for new CMBs after cardiac surgery.

**Clinical Trial Registration—:**

URL: http://www.isrctn.com. Unique identifier: 66022965.

## Abstract 2

Supplemental Digital Content is available in the text.

The presence of cerebral microbleeds (CMBs) has been demonstrated using magnetic resonance imaging (MRI) in the brains of patients with cardiovascular risk factors, cognitive deterioration, small vessel disease, and dementia.^1^ The most reliable MRI method for the detection of CMBs is susceptibility-weighted imaging (SWI), owing to its sensitivity to the presence of iron-storage complex hemosiderin,^2–4^ which is deposited in abundance following hemorrhage.^3^ SWI imaging has been found to be almost 6× more sensitive to microhemorrhagic events than a conventional T2-weighted gradient-echo sequence.^5^

CMBs may occur in the presence of amyloid deposits in the walls of cerebral blood vessels (cerebral amyloid angiopathy [CAA]), as well as with chronic hypertension, diffuse traumatic axonal injury, and during critical illness, among other causes.^6^ CMBs consequently mark the presence of diffuse vascular, traumatic, and neurodegenerative brain damage.

The results of recent studies investigating the role of CMBs in cognitive impairment have been inconsistent. The Rotterdam Scan Study reported that the presence of CMBs increases with age in 18% of people aged 60 to 69, and in 39% of those >80 years.^7^ Healthy subjects with CMBs showed reduced motor speed and information processing^8^ as well as shortened attention, indicating a worsening outcome as assessed by the Mini-Mental State Examination.^9,10^

A small case study reported gradient-echo–detected CMBs after cardiopulmonary bypass (CPB) in 6 children. All cases showed multiple microbleeds (>3 CMBs) situated in the cortical or lobar regions.^11^ Another study of 23 pediatric cardiac surgery patients receiving CPB reported the incidence of new CMBs postsurgery to be 90%.^12^ However, the prevalence, location, and frequency of CMBs in adult cardiac surgery, in relation to perioperative factors and cognitive status, is unknown.

CPB has long been considered a major risk factor for neurological injury after cardiac surgery. Injury to the brain after cardiac surgery is not restricted to hemorrhagic events but incorporates a range of neurological harmful effects including hypoxia, ischemia, and stroke.^13,14^ A study by Michałowska et al,^15^ reported SWI lesions in patients undergoing off-pump (n=36) and on-pump (n=15) cardiac surgery. The authors reported that patients undergoing on-pump surgery had more SWI lesions compared with the off-pump group, though the comparison was not controlled for covariates such as age, sex, and perioperative risk factors.

Modern MRI techniques, such as SWI, enable confident identification of the number and spatial distribution of CMBs. This study aimed to estimate the burden of new CMBs in adult cardiac surgery patients relative to intraoperative factors and cognitive status. CMBs are not a rare observation in the aging population; therefore, it is necessary to assess whether physiological stresses associated with cardiac surgery give rise to an additional burden of new CMBs and cognitive decline, and if so, what factors are associated with each of these outcomes.

## Methods

### Patients

The authors declare that all supporting data are available within the article (and in the online-only Data Supplement). All patients needing on-pump coronary artery bypass graft or valve surgery at Leicester Glenfield Hospital were eligible for inclusion in this study. Patients were excluded if they had contraindications to MRI (eg, a cardiac pacemaker), or if their first language was not English, because of the neuropsychological tests containing a language component. Patient characteristics available for analysis as part of this study included age, sex, educational level, smoking status, hypertension, hypercholesterolemia, and ischemic heart disease. Written informed consent was provided by all patients following a protocol approved by the University Hospitals of Leicester NHS Trust and Derbyshire Research Ethics Committee (REC reference: 10/H0401/78). The study was sponsored by the University of Leicester.

### Anesthetic and Surgical Procedures

All patients received routine perioperative care, and there were no explicit alterations to standard surgical practice. Cold crystalloid cardioplegia was used, and anesthetic management consisted of a combination of propofol, midazolam, isoflurane, and fentanyl. Body temperature was measured every 3 minutes with a nasal pharyngeal temperature probe. Nonpulsatile CPB was implemented with a nonocclusive roller pump to achieve a target CPB perfusion pressure >50 mm Hg. The CPB circuit contained a 38 μm arterial line filter and a membrane oxygenator. Arterial blood pressure targets during surgery were based on usual clinical practice.

### Neuropsychological Assessment

Neuropsychological tests were performed 1 to 2 weeks before surgery and 6 to 8 weeks postoperatively at the same time points as the MRI scans. Our test battery included 2 tests recommended by the Statement of Consensus on Assessment of Neurobehavioral Outcome after Cardiac Surgery^16^: the Grooved Pegboard Test^17^ and the Trail Making Tests (parts A and B).^18^ In addition, the Wechsler Abbreviated Scale of Intelligence^19^ and the Wechsler Memory Scale-Third Edition^20^ were administered. Patients also completed a Hospital Anxiety and Depression Scale questionnaire.^21^ These tests were selected to allow pairwise comparison of before and after scores, as well as being accompanied by well-characterized normative data in age-matched healthy subjects (analyzed via *z*-score analysis). Neuropsychological assessments were performed by a trained assessor who was blinded to the imaging data.

### Magnetic Resonance Imaging

MRI was performed 1 to 2 weeks before surgery and 6 to 8 weeks postoperatively at the same time points as the neuropsychological assessment. All MRI examinations were conducted using a 3T whole-body scanner (Magnetom Skyra; Siemens Medical, Erlangen, Germany). Scans were performed in the following order: 3-plane localizer; diffusion-weighted sequence; time of flight MR angiography; SWI; and fluid-attenuated inversion recovery. For the SWI, the k-space matrix was 256×213 reconstructed to 256×224, field of view was 240 mm×210 mm, slice thickness was 3 mm, repetition time/echo time were 1290/20 ms with a flip angle of 60 degrees. The total imaging time was ≈30 minutes. To distinguish new CMBs from old CMBs, SWI images were presented to a qualified neuroradiologist (A. Cox) who was blinded to the results of the neuropsychological assessments. Fluid-attenuated inversion recovery images were also presented for detection of new ischemic changes.

### Identification of CMBs

**Figure 1.**
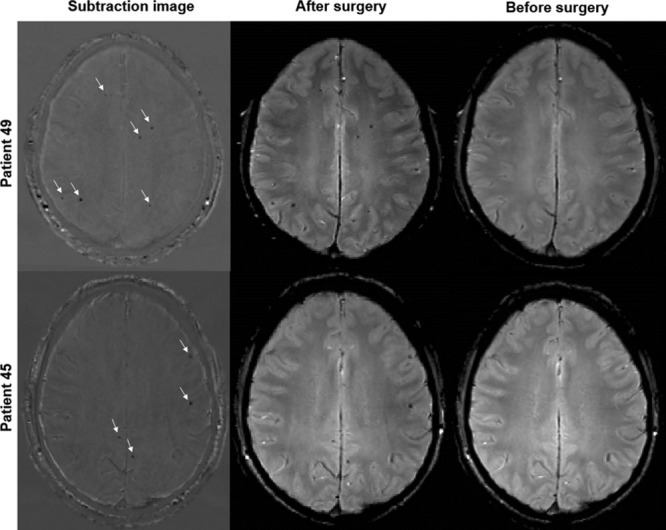
Comparison of susceptibility-weighted imaging images before and after cardiac surgery. Registration and subtraction of the magnetic resonance imaging data were performed using JIM software (Xinapse Ltd) to confidently distinguish new cerebral microbleeds (CMBs) from preexisting CMBs, providing the number and location of CMBs.

SWI images were examined by a neuroradiologist using the Brain Observer Microbleed Rating Scale to aid identification and location of CMBs.^22^ CMBs were identified as defined focal areas of low signal on SWI, <10 mm in diameter. Symmetrical areas of basal ganglia calcification, flow voids from blood vessels, and low signal from adjacent bone were excluded. New CMBs were then characterized through registration and subtraction of pre- and postoperative SWI images using the medical image analysis package Jim (version 7, Xinapse Systems, Colchester, United Kingdom; http://www.xinapse.com; Figure 1).^23^ Images were assessed for the location and number of both new and preexisting CMBs, which were delineated using a semiautomatic contouring technique. To aid visualization of the distribution of new CMBs, postoperative SWI images were registered to a standard MRI brain atlas,^24^ and new CMBs were segmented and displayed using the atlas as reference for the 3-dimensional (3D) display.

### Identification of CAA

Probable CAA was characterized by the neuroradiologist using the Diagnosis of Cerebral Amyloid Angiopathy: Evolution of the Boston Criteria.^25^ Information was sourced from clinical data, and MRI is demonstrating multiple hemorrhages (intracerebral hemorrhages, CMBs) restricted to cortical, lobar, or cortical/sub-cortical regions (including cerebellar hemorrhage), or single lobar, cortical, or cortical/subcortical hemorrhage and superficial siderosis (focal or disseminated).

### Statistical Analysis

Data are presented as mean [±SD] unless stated otherwise. Statistical analyses were performed using the SPSS software package (Statistical Product and Service Solutions, SPSS, version 25; SPSS Inc). Tests for normality were performed using the Kolmogorov-Smirnov test. Differences with a *P* value of <0.05 were considered to be statistically significant. Clinical demographic variables between patients with and without new CMBs were compared using Fisher test, a *t* test, or χ^2^ test as appropriate. Interactions between cognitive status and the presence or absence of CMBs were examined using ANCOVA. Before data analysis, checks for any outliers of abnormities were performed. Two separate binomial logistic regression models were fitted for each perioperative variable which included the age of the patient, sex, hypertension, CPB hematocrit levels, CPB time, mean arterial pressure, and the number of new CMBs. Model outputs are reported with their corresponding odds ratios, the 95% CIs for the odds ratio, and associated *P* values. Where applicable, variables were adjusted for age, sex, and educational level.

Individual neuropsychological test scores were converted to *z* scores through comparison with published data describing the mean (*X*) and SD of test scores measured from a population of healthy subjects:


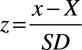
(1)

Postoperative *z*scores were then subtracted from preoperative *z*scores to calculate the pairwise change in *z*score; a significant decline in cognition was assumed if there was a drop in *z*score of >1 SD from baseline. For timed tests (Trail Making A/B and Grooved Pegboard tests), the sign of the *z*score was reversed, such that poorer performance corresponded to a negative *z*score. In addition to calculating the *z*score change for each individual test, *z*scores were summed and averaged to quantify the overall cognitive performance of each patient as a composite cognitive performance score.^26^

## Results

Of the 103 patients recruited in the study, 20 did not receive a preoperative MRI scan because of scheduling difficulties, 4 were unable to receive a postoperative scan (fitted pacemaker), and 4 withdrew from the study. Complete pre- and postoperative MRI and cognitive test data were analyzed from the remaining 75 patients (70 men; 63±10 years). Diffusion-weighted images were also examined alongside SWI images. Because diffusion-weighted imaging is sensitive only to acute ischemic lesions, the length of time from surgery to the postoperative MRI scan precluded any conclusions being drawn about the relationship between perioperative ischemia and new CMBs. A table summarizing all data relating to this study is provided in Tables I and II in the online-only Data Supplement.

After surgery, new CMBs were identified using SWI MRI in 76% (57/75) of patients. Fourteen patients (24%) had at least 1 new CMB, 26 patients (46%) had 2 to 5 new CMBs, and 17 patients (30%) had >5 new CMBs. The highest reported number of new CMBs was observed in patient 38 who had 43 new CMBs. The CPB time for this patient was 254 minutes compared with the cohort average of 90 minutes: SD, 49 (full details for all patients can be found in Tables I and II in the online-only Data Supplement). The prevalence of preexisting CMBs was small in comparison to new CMBs. In this cohort, 36% (27/75) had preexisting CMBs, of which 67% (18/27) had only 1 preexisting microbleed, 22% (6/27) had 2 to 3 preexisting CMBs, and 11% (3/27) had 6 to 7 preexisting CMBs. All preexisting CMBs were observed in the postoperative SWI scans.

Comparison between the groups with and without new CMBs showed significant differences in the new CMBs group for type of procedure (coronary artery bypass graft versus valve; χ^2^ test, *P*=0.007), and CPB time (97 versus 68 minutes; *t* test, *P*=0.003). There were borderline associations with lower hematocrit (mean hematocrit: 27% versus 30%; *t* test, *P*=0.045), and number of preexisting CMBs (0.82 versus 0.32; *t* test, *P*=0.021; Table 1). All 9 patients with CAA had new CMBs postcardiac surgery (Table 1). The volume of new ischemic lesions after cardiac surgery was also significantly higher in patients with new CMBs (82 versus 11 cm^3^; *t* test, *P*=0.032).

**Table 1.**
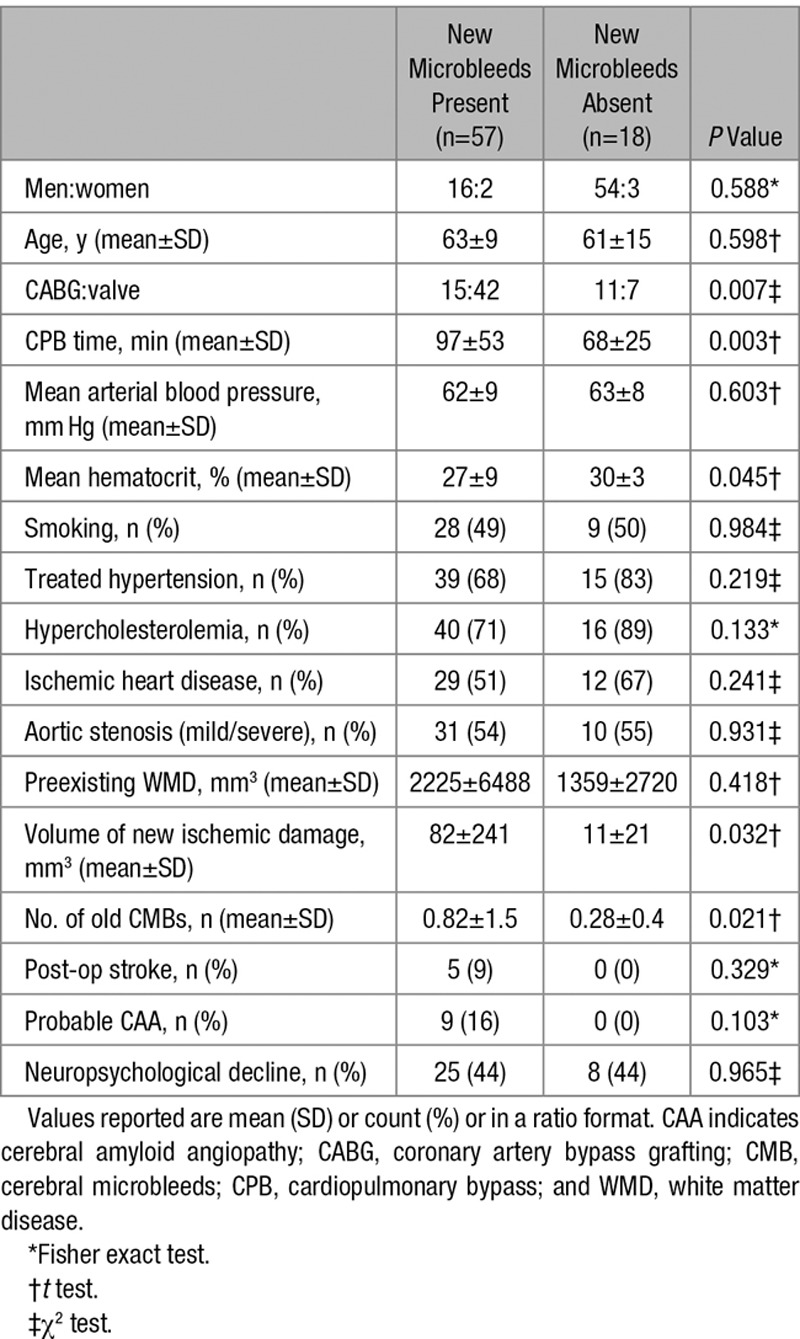
Clinical and Demographic Characteristics of Patients With and Without New CMBs After Cardiac Surgery

**Figure 2.**
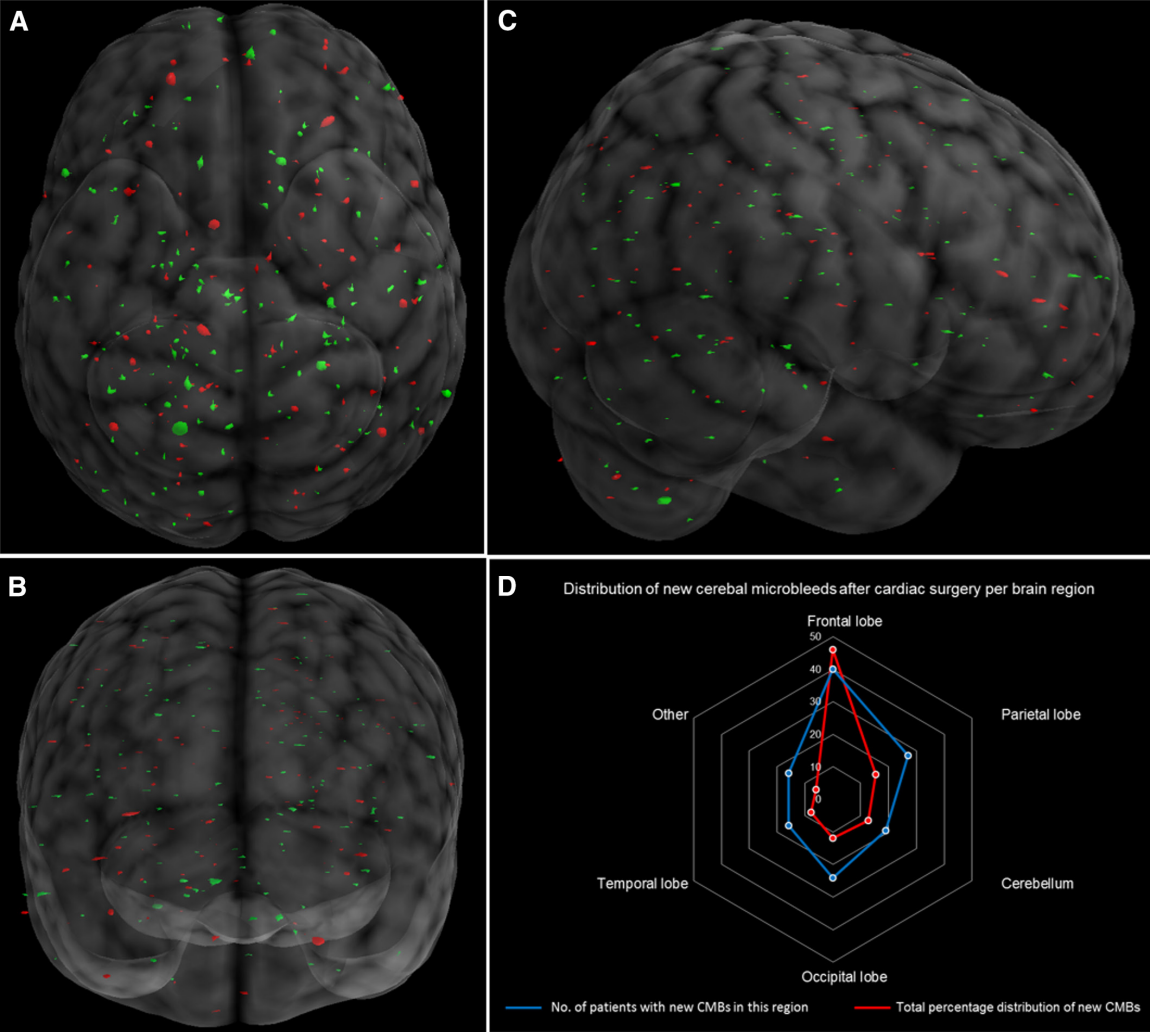
The spatial distribution of new cerebral microbleeds (CMBs) after cardiac surgery, compiled from the combined data of 57 patients with (green) and without (red) postoperative cognitive decline. Microbleeds are highlighted against the background of a standard atlas image. **A**, Superior, (**B**) coronal view, and (**C**) lateral view. **D**, Radar plot showing the distribution of new CMBs (blue line); number of patients with new CMBs in this region (red line); and total percentage distribution of new CMBs per brain region.

A 3D illustration of the overall distribution of new CMBs, from the 57 patients with changes after cardiac surgery, is presented in Figure 2. New CMBs after cardiac surgery in patients with cognitive decline are shown in red and without cognitive decline in green. The majority of new CMBs were located in the frontal lobe (46%) followed by the parietal lobe (15%), cerebellum (13%), occipital lobe (12%), temporal lobe (8%), hippocampal (1.4%), putamen/globus pallidus (1.4%), thalamus (1%), and the midbrain (1%). A detailed presentation of the location and distribution of new CMBs after surgery is provided in Table III in the online-only Data Supplement.

A decline in neuropsychological test performance of >1 SD after surgery in ≥1 tests was noted in 33 (44%) of 75 patients. Five patients (15%) declined in >2 tests. Cognitive regions for decline were associated with psychomotor function (54% [18]), executive function (39% [13]), working memory (18% [6]), and long-term memory (9% [3]). Most patients had typical levels of depression and mild levels of anxiety on the Hospital Anxiety and Depression Scale at preoperative testing (mean [SD]; anxiety, 6.1 [3.6]; depression, 3.8 [3.0]), which were stable or reduced after surgery (anxiety, 3.8 [2.5]; depression, 2.8 [3.1]). Decline in neuropsychological performance was therefore not attributable to heightened anxiety or depression.

**Figure 3.**
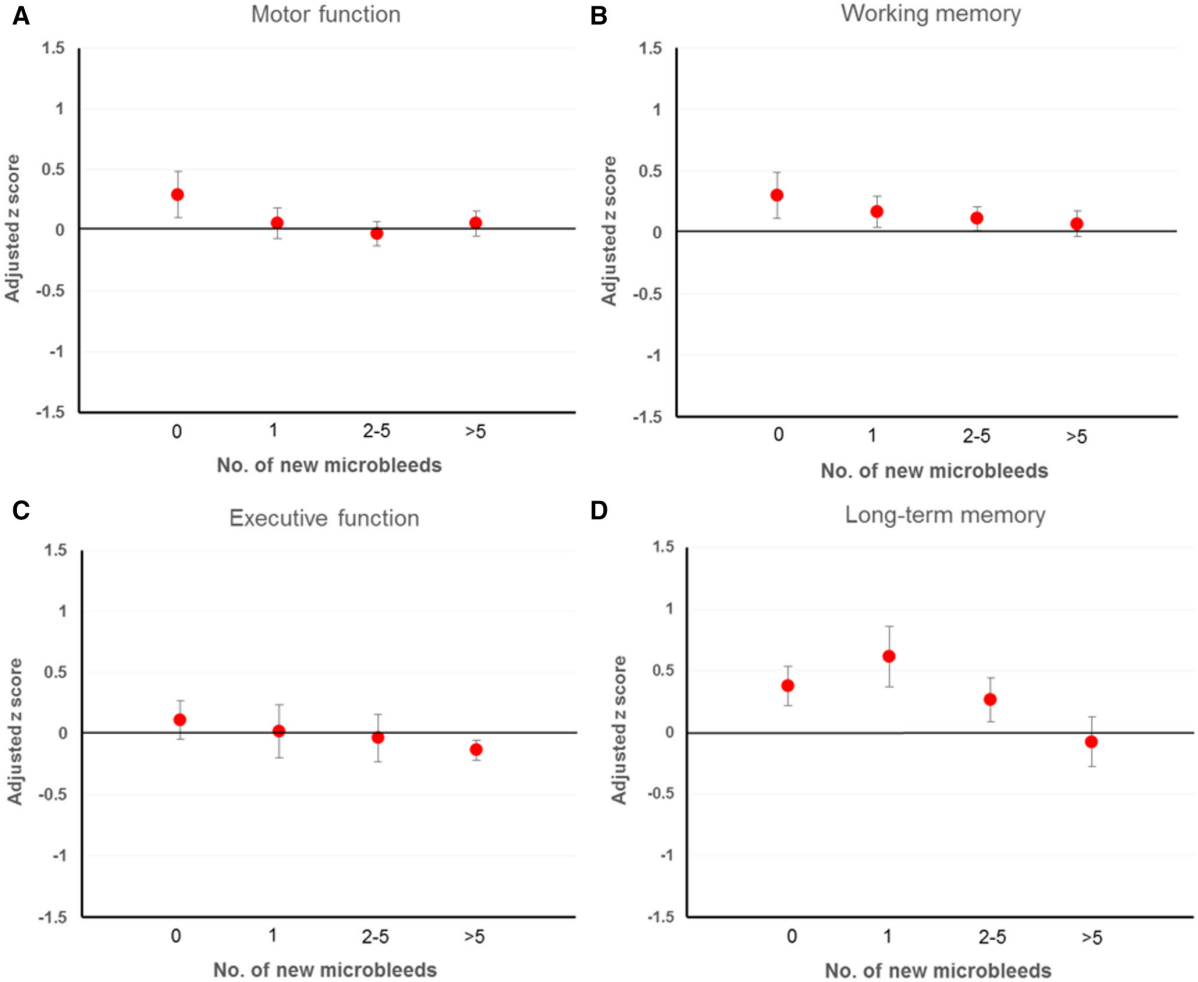
Age, sex, and educational level adjusted average *z* scores are presented for cognitive outcomes in specific domains: (**A**) motor function, (**B**) working memory, (**C**) executive function, and (**D**) long-term memory. Patients were grouped by cerebral microbleed (CMB) count (0 CMBs, n=18; 1 CMBs, n=14; 2–5 CMBs, n=26; >5 CMBs, n=17) to calculate the average *z* score of the group. Error bars represent 95% CIs on the mean.

The 4 major neuropsychological domains (executive function, psychomotor function, working memory, and long-term memory) were studied in relation to the development of new CMBs. Adjusted *z*scores were lower for all 4 cognitive domains in the group of patients with new CMBs; however, there were no significant differences between subjects with and without CMBs after controlling for age and sex (Table 2). The greatest decline in mean postoperative *z*scores between groups with and without new CMBs were observed in executive function tasks (mean [SD]: −0.03 [0.10] versus 0.21 [0.19], ANCOVA, *P*=0.24) and psychomotor function (0.02 [0.07] versus 0.26 [0.19], ANCOVA, *P*=0.07). The number of new CMBs were separated into 3 groups: (i) 1 CMB, (ii) 2 to 5 CMBs, and (iii) >5 CMBs (Figure 3). Marginal decreases in average *z*scores with increasing number of CMBs were noted for working memory, executive function, and long-term memory, but given the wide variations observed in our cohort, these did not prove to be statistically significant (Table 2). Our analysis did not provide evidence for a trend between multiple new CMBs and declining neuropsychological test scores in any of the domains tested.

**Table 2.**
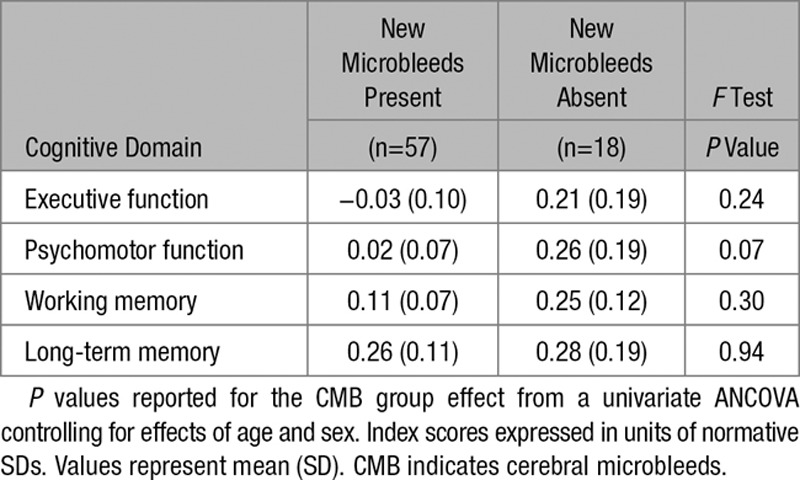
Comparison of Cognition in Cases With and Without New CMBs After Cardiac Surgery

The contribution of various perioperative risk factors during cardiac surgery against the outcome of new CMBs and neuropsychological impairment was explored using 2 separate logistic regression models with outcomes of (i) new microbleeds and (ii) neuropsychological decline. Covariates for the model included age, sex, CPB time, hypertension, mean arterial pressure, hematocrit, and number of new CMBs. CPB time was the only significant predictor for new CMBs during cardiac surgery (1.02; 95% CI, 1.00–1.05; *P*=0.04) suggesting a ≈2% increase in the odds of acquiring new CMBs for every minute of bypass time over 80 mins (Table 3). Age was the only significant predictor of cognitive impairment postcardiac surgery (1.07; 95% CI, 1.01–1.13; *P*=0.01), with the risk of cognitive decline increasing by ≈7% per year of age over 63 years (Table 3). While hypertension and mean arterial pressure were also investigated, there was no significant association of blood pressure parameters with new CMBs (Table 3).

**Table 3.**
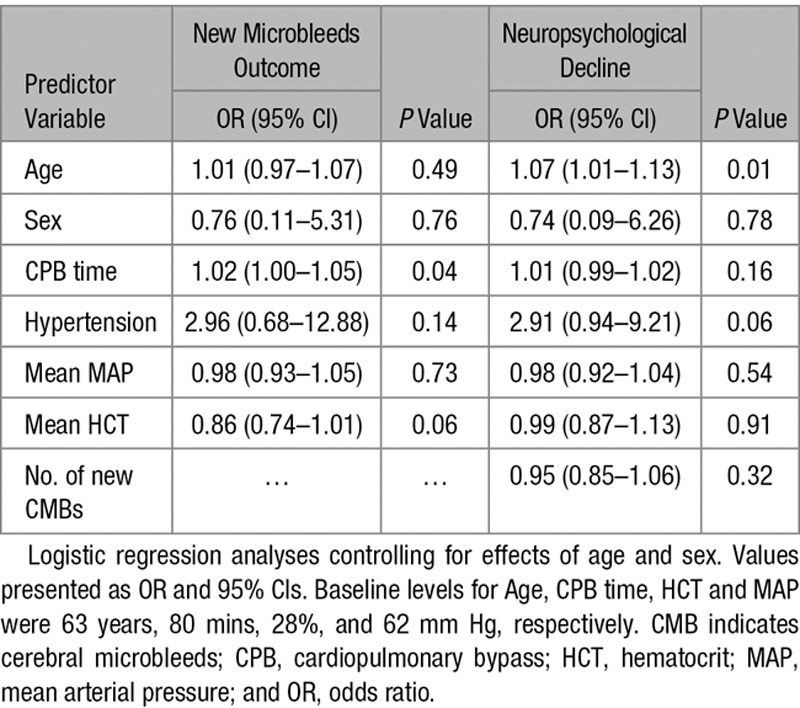
Binomial Logistic Regression Analyses for Outcomes of New CMBs and Neuropsychological Decline

## Discussion

SWI is a powerful MRI sequence that is almost 6× more sensitive to microhemorrhagic events than conventional T2-weighted gradient-echo.^4^ SWI’s higher sensitivity to levels of hemosiderin, an iron-storage complex found in abundance after hemorrhagic events^2–4^ allows for better detection of CMBs.

### Comparison With Pediatric Studies

In this present study, 76% of patients had new CMBs after cardiac surgery with 51% having multiple CMBs. The prevalence of new CMBs after heart surgery in adults was lower than previously reported after pediatric surgery. The study by Kim et al^12^ investigated CMBs using SWI in 23 pediatric patients with heart failure who underwent surgery using CPB, against 13 pediatric patients with no CPB (ie, no surgery). The authors reported significantly higher numbers of CMBs in the surgical group compared with the nonsurgery group (*P*<0.001). The authors concluded that exposure to CPB increases the prevalence and burden of CMBs among pediatric patients with heart failure.

### CPB Machine

There are several perioperative risk factors associated with neurological damage during cardiac surgery.^27^ CPB time tends to increase with complexity of the surgery and has previously been linked to neurological complications such as acute encephalopathy, stroke, and cognitive decline.^8,10^ During CPB, air emboli can also be introduced into the CPB circuit, detected using transcranial Doppler ultrasound.^28^ Our logistic regression model suggests that the length of CPB time may be a significant predictor for the development of new CMBs after cardiac surgery (odds ratio, 1.02; 95% CI, 1.00–1.05; *P*=0.04). This implies an increase in the odds of acquiring new CMBs of between 0% and 5% for every minute of bypass time over 80 mins. Although the *P* value is borderline and the CI includes 1, even a very small increase in risk associated with increased bypass time, when measured on a minute-by-minute basis, has the potential to accumulate significantly over the course of surgery. New CMBs were therefore associated with significantly longer CPB times (*P*=0.003; Table 1).

### Cognitive Outcome

Previous trials that have related CMBs to cognition in a stroke population and in normal aging have produced conflicting results. The majority of studies in healthy aging populations reported an association between neuropsychological outcome and CMBs.^9,10^ Studies of patients with symptomatic cerebrovascular disease and CMBs have yielded less consistent results.^29,30^ A study by Patel et al^30^ looked at the relationship between CMBs and neurocognitive performance in a small vessel disease cohort. Another study by Pettersen et al^31^ looked at neurocognitive performance and CMBs in patients with Alzheimer disease. The authors of both studies were unable to find a link between neuropsychological performance and CMBs.

The location of CMBs may be more significant than the number of CMBs in predicting neuropsychological sequelae. In the study by Pettersen et al,^31^ stroke patients with a decline in executive function had more CMBs in the basal ganglia compared with a control group. However, in a normal aging cohort, thalamic CMBs were linked with worse coordination on the Mini-Mental State Examination, which suggests a decline in executive function and visuospatial abilities.^9^ In a large cohort of patients with cerebral small vessel disease, neuropsychological deficits were associated with CMBs in the frontal and temporal lobes,^29^ whereas another study showed that the number of basal ganglia, thalamic, and frontal CMBs did not predict cognitive deficits after controlling for other MRI parameters.^30^ A recent study by Xiong et al^32^ studied CMBs and their association with cognition in patients with CAA. The authors found the presence of CMBs to be associated with impaired executive function (*P*=0.036) and processing speed (*P*=0.020); however, no significant difference was observed in global neuropsychological performance in patients with and without CMBs. Although we report neurocognitive decline in 46% of our patients postcardiac surgery, this did not seem to be related to the observed number of new perioperative CMBs.

### Probable CAA Diagnosis and New CMBs

CAA is characterized by extracellular deposition of amyloid-β around cerebral arteries and capillaries, leading to an increased risk of neurological damage, such as hemorrhagic events (lobar and subarachnoid).^25^ This is a condition of increasing clinical and mechanistic importance, as patients with sporadic CAA have a higher mortality and are at risk of recurrent hemorrhage.^33^ The recent study by Xiong et al^32^ also found that the presence of CMBs predicted dementia conversion in patients with CAA. The risk of damage to the vasculature because of the CPB machine during cardiac surgery may predispose some patients to receive new CMBs. Using the Evolution of the Boston Criteria for Diagnosis of Cerebral Amyloid Angiopathy,^25^ the neuroradiologist diagnosed a total of 9 patients with probable CAA. All 9 patients were reported to have developed new CMBs after cardiac surgery. Although the subpopulation of CAA in our sample was small (12%), this illustrates the vulnerability of patients with probable CAA to cerebral hemorrhagic damage when undergoing cardiac surgery. Further studies with a larger sample population of patients with CAA should be performed to elucidate the risk of cerebral vascular damage in this subgroup of patients when undergoing cardiac surgery.

### Hematocrit and New CMBs

Mean [SD] hematocrit levels during CPB for all 75 patients in this study was 28% [3.9]. Previous studies have reported that hematocrit levels that drop below 22% may be a risk factor for perioperative neurological damage.^14^ To the best of our knowledge, this is the first study to report hematocrit levels in relation to new CMBs during cardiac surgery. In this cohort, patients with new CMBs had slightly lower hematocrit level (27%) compared with the group of patients with no new CMBs (30%; *t* test, *P*=0.04). The volume fraction of red blood cells in a capillary affects the degree to which platelets are promoted to marginate to near a vessel wall to allow the formation of blood clots.^34^ Previously, an in vitro study investigating the relationship between red blood cell hematocrit levels and platelet adhesion activity was performed. This involved passing flowing blood through a microfluidic channel coated with type 1 collagen to determine the rate to which platelets adhered to the wall. The study reported that the rate of platelet adhesion differs significantly with hematocrit levels, with a relative decrease in platelet activity as hematocrit levels fall.^34^ In this study, we observed a slightly lower hematocrit in patients with new CMBs (*t* test, *P*=0.04). Our logistic regression model suggested a relationship between hematocrit levels and new CMBs of borderline significance (odds ratio, 0.86; 95% CI, 0.74–1.01; *P*=0.06), estimating that a 1 % increase in hematocrit above 28% reduces the risk of CMBs by ≈14%. A larger sample size would be required to confirm this finding.

### Limitations

The use of anticoagulation/antithrombotic therapy may have played an important role in the observed results and may have been useful as a predictor of new CMBs. Unfortunately, in this study, these data were not recorded. This would be of significant interest in future trials. Furthermore, this study would be strengthened with one or more additional neuroradiologists to read the MRI scans so that interreader variability for new and existing CMBs could be reported.

### Conclusions

This study confirmed that CMBs and cognitive decline are common after cardiac surgery; 76% of patients had new CMBs, and 44% experienced cognitive decline. The presence of new CMBs postcardiac surgery is high in comparison to preexisting CMBs with a 7.6-fold increase in CMBs postcardiac surgery. Length of CPB time was a significant predictor of new CMBs. Levels of hematocrit were significantly lower in patients with new CMBs and patients with preexisting CMBs were more likely to have new CMBs after cardiac surgery. The presence of new CMBs was not found to be related to neuropsychological impairment, although some trends were observed that should be investigated in a larger study. Longitudinal studies may provide more robust information about whether CMBs are indeed markers of future cognitive decline. Studying a larger population would also allow the relationship between hematocrit levels and new CMBs to be further investigated.

## Acknowledgments

We thank the participants, the theater staff of the Cardiothoracic Surgery Unit, and the MRI radiographers at the Leicester Glenfield Hospital. Dr Chung thanks Mr Morgan Lillie for reviewing the Radiologist reports relating to this study.

## Sources of Funding

This study was funded by the British Heart Foundation (FS/10/46/288350) and the Engineering and Physical Sciences Research Council (EP/L025884/1). Dr Patel was funded by an NIHR Leicester Biomedical Research Centre doctoral studentship.

## Disclosures

Dr Horsfield is an employee and stockholder of Xinapse Systems Ltd.

## Supplementary Material
