# Supplementary material for: Perioperative Cerebral Microbleeds After Adult Cardiac Surgery
Source: Stroke. 2018 Dec 21;50(2):336–43. doi: 10.1161/STROKEAHA.118.023355 (PMC6354910; doi:10.1161/STROKEAHA.118.023355)
Supplement: Supplementary file 1 [file str-50-336-s001.pdf]

SUPPLEMENTAL MATERIAL

Table I: Detailed summary of clinical demographics for all 75 patients. Patients marked with * had perioperative stroke diagnosed clinically. CAA indicates cerebral amyloid angiopathy.

Patient no.	Age	Gender	Procedure	Smoking	Ischaemic heart disease	Hypertension	Hypercholesterolemia	Aortic stenosis	Probable CAA
**1**	45	F	Valve	Yes	No	No	No	Yes	No
**2**	57	M	CABG	No	Yes	Yes	Yes	No	No
**3**	72	M	CABG	Yes	Yes	Yes	Yes	No	No
**4**	75	M	Valve	No	Yes	Yes	Yes	Yes	No
**5**	76	M	CABG	Yes	Yes	Yes	Yes	No	No
**6**	64	M	CABG	Yes	Yes	No	Yes	Yes	No
**7**	65	M	Valve	No	Yes	Yes	Yes	Yes	No
**8**	59	M	Valve	No	Yes	Yes	Yes	No	No
**9**	59	M	Valve	Yes	Yes	Yes	Yes	Yes	No
**10**	79	M	Valve	No	Yes	Yes	No	Yes	No
**11**	57	M	Valve	No	No	Yes	No	Yes	Yes
**12**	50	M	Valve	Yes	No	No	No	Yes	No
**13**	65	M	Valve	No	No	No	No	Yes	No
**14**	40	F	Valve	No	Yes	Yes	Yes	Yes	No
**15**	59	M	Valve	Yes	Yes	Yes	Yes	Yes	No
**16**	65	M	Valve	Yes	Yes	No	Yes	Yes	No
**17**	57	M	CABG	No	Yes	Yes	No	No	No
**18**	68	M	Valve	Yes	No	No	Yes	Yes	No
**19**	32	M	CABG	No	Yes	Yes	No	No	No
**20**	75	M	Valve	Yes	Yes	Yes	Yes	No	No
**21**	69	M	Valve	Yes	No	Yes	Yes	Yes	No
**22**	72	M	Valve	Yes	Yes	Yes	Yes	Yes	Yes
**23**	49	M	Valve	Yes	No	Yes	No	Yes	No
**24**	67	M	CABG	Yes	Yes	No	Yes	No	No
**25**	61	M	Valve	Yes	No	Yes	Yes	No	Yes
**26**	60	M	Valve	No	No	Yes	Yes	Yes	Yes
**27**	65	M	Valve	No	No	No	No	No	No
**28**	62	M	Valve	No	No	No	No	No	No
**29**	61	M	CABG	Yes	Yes	Yes	Yes	Yes	No
**30**	61	M	CABG	Yes	Yes	Yes	Yes	Yes	No

**31**	66	M	Valve	Yes	Yes	Yes	Yes	Yes	No
**32**	74	M	Valve	No	No	Yes	Yes	No	No
**33**	64	M	Valve	No	Yes	Yes	No	No	No
**34**	60	M	CABG	No	No	Yes	Yes	No	Yes
**35**	41	M	Valve	No	No	Yes	Yes	No	No
**36**	55	M	Valve	Yes	No	No	Yes	Yes	No
**37**	71	F	Valve	Yes	Yes	No	Yes	Yes	No
**38**	61	M	Valve	No	No	Yes	Yes	No	No
**39**	59	M	Valve	No	No	No	No	Yes	No
**40**	46	M	Valve	Yes	No	Yes	Yes	Yes	No
**41**	32	M	Valve	No	No	No	No	No	No
**42**	65	M	CABG	No	Yes	Yes	Yes	Yes	No
**43**	64	M	Valve	No	No	No	No	No	Yes
**44***	71	F	CABG	Yes	Yes	Yes	Yes	No	No
**45**	80	M	Valve	No	Yes	Yes	No	Yes	No
**46***	64	M	Valve	No	No	No	No	No	No
**47***	50	M	Valve	Yes	Yes	No	Yes	Yes	No
**48**	71	M	CABG	No	No	Yes	Yes	No	No
**49**	76	M	CABG	Yes	No	Yes	Yes	No	No
**50**	68	M	CABG	Yes	Yes	Yes	Yes	No	No
**51**	69	M	CABG	No	Yes	Yes	Yes	Yes	No
**52**	63	M	CABG	No	No	Yes	Yes	No	No
**53***	66	M	CABG	No	Yes	Yes	Yes	No	No
**54**	77	M	CABG	Yes	No	Yes	Yes	Yes	Yes
**55**	57	M	CABG	Yes	Yes	Yes	Yes	Yes	No
**56**	59	M	Valve	No	Yes	Yes	Yes	Yes	No
**57**	72	M	Valve	Yes	Yes	Yes	Yes	Yes	No
**58***	57	M	Valve	No	No	No	No	No	No
**59**	65	M	Valve	Yes	No	No	No	Yes	No
**60**	56	M	Valve	Yes	No	No	Yes	No	No
**61**	78	M	Valve	Yes	Yes	Yes	Yes	Yes	No
**62**	54	M	Valve	No	No	Yes	Yes	Yes	No
**63**	77	M	CABG	No	Yes	No	Yes	No	No
**64**	69	M	Valve	Yes	No	Yes	Yes	Yes	No
**65**	65	M	Valve	Yes	No	Yes	Yes	No	Yes
**66**	71	M	Valve	Yes	No	Yes	Yes	Yes	No
**67**	72	M	CABG	No	Yes	No	Yes	No	No
**68**	72	M	Valve	No	Yes	Yes	No	Yes	Yes
**69**	68	M	Valve	No	Yes	Yes	Yes	Yes	No
**70**	54	M	Valve	Yes	Yes	Yes	Yes	Yes	No
**71**	63	M	Valve	No	Yes	Yes	Yes	Yes	No

**72**	56	M	CABG	No	Yes	Yes	Yes	No	No
**73**	53	M	CABG	Yes	No	Yes	Yes	No	No
**74**	62	F	CABG	No	Yes	Yes	Yes	No	No
**75**	76	M	CABG	Yes	No	Yes	Yes	No	No

Table II: Intra-operative and post-operative outcomes for all 75 patients. CPB indicates cardiopulmonary bypass. HCT indicates haematocrit. BP indicates blood pressure.

Patient no.	CPB Time, (mins)	Mean Arterial BP (mm Hg)	Mean HCT (%)	No. of old microhaemorrhages	No. of new microhaemorrhages	Volume of pre-existing lesions (mm^3)	Volume of new lesions (mm^3)
**1**	76	74.4	26.1	0	1	322	0
**2**	84	71.9	29.1	1	1	0	0
**3**	82	54.8	23.9	0	2	2656	0
**4**	139	61.9	26.5	1	8	1974	241
**5**	55	55.2	24.5	0	2	17568	0
**6**	93	61.0	29.4	0	0	387	0
**7**	163	76.5	24.5	0	2	742	621
**8**	87	55.1	22.2	0	4	1098	0
**9**	76	64.8	23.9	0	2	0	0
**10**	100	52.2	31.5	0	1	6446	0
**11**	122	65.0	28.3	3	3	5490	0
**12**	94	46.5	22.4	0	10	0	0
**13**	123	58.8	26.4	0	8	650	0
**14**	92	53.3	28.4	0	0	21	47
**15**	109	55.2	24.1	0	3	338	0
**16**	95	60.4	37.6	0	1	672	0
**17**	32	52.4	33.1	0	1	1037	0
**18**	103	59.4	27.5	1	3	711	44
**19**	63	59.3	31.0	1	0	586	0
**20**	78	57.4	26.0	0	4	3655	0
**21**	111	68.5	33.1	0	0	0	0
**22**	88	74.6	25.4	2	5	0	0
**23**	84	79.9	22.3	0	6	1736	13
**24**	46	68.9	27.9	1	3	0	0
**25**	107	76.9	25.3	3	9	0	0
**26**	273	78.9	23.4	6	16	190	108
**27**	185	70.3	22.2	1	8	550	0
**28**	80	56.0	27.8	1	5	575	0
**29**	51	54.3	31.0	0	0	0	0
**30**	77	62.8	31.0	0	0	958	17
**31**	80	45.7	27.8	0	16	0	0
**32**	53	64.6	33.1	0	7	1231	37
**33**	80	59.6	31.5	1	1	768	0
**34**	39	54.8	31.0	3	3	0	0
**35**	153	52.8	31.0	1	23	0	0

**36**	71	51.8	28.7	0	10	1876	0
**37**	66	60.7	25.1	1	5	7055	309
**38**	254	54.1	28.7	0	43	0	0
**39**	89	62.6	37.6	0	4	0	28
**40**	80	68.5	27.6	1	0	3416	26
**41**	40	66.0	29.4	0	0	0	0
**42**	48	83.4	28.4	1	0	0	0
**43**	77	65.3	28.3	7	3	914	5
**44**	40	80.5	22.2	0	4	620	1383
**45**	65	51.6	21.4	1	8	260	167
**46**	64	59.7	27.4	0	11	1110	81
**47**	63	55.0	28.3	0	8	3516	389
**48**	48	74.6	27.9	1	0	772	0
**49**	45	55.2	35.9	0	0	2649	0
**50**	54	55.7	22.2	0	0	427	0
**51**	43	53.1	26.1	1	1	0	51
**52**	66	80.7	33.1	0	1	186	63
**53**	95	59.6	27.9	0	2	743	175
**54**	75	61.8	25.2	2	1	4078	0
**55**	62	62.6	27.9	0	3	1076	0
**56**	71	80.9	35.9	0	0	0	0
**57**	271	61.4	29.7	1	8	0	0
**58**	44	55.7	26.1	0	1	911	979
**59**	120	62.6	26.5	0	2	602	15
**60**	119	63.1	22.5	0	5	0	0
**61**	62	59.5	25.7	0	0	1073	0
**62**	87	58.3	34.3	0	1	0	0
**63**	54	57.4	31.8	0	0	0	0
**64**	111	60.9	36.4	0	1	3790	0
**65**	219	60.9	25.3	2	11	1146	0
**66**	90	60.5	36.4	1	2	1146	0
**67**	53	62.0	32.6	0	2	0	0
**68**	92	69.8	29.6	6	2	0	0
**69**	87	64.9	30.5	1	0	27950	67
**70**	112	56.5	26.8	0	1	0	0
**71**	72	86.4	28.2	0	3	0	0
**72**	74	63.2	29.6	0	1	0	0
**73**	62	69.1	29.3	0	2	0	0
**74**	35	62.7	26.4	0	0	750	0
**75**	122	60.3	31.8	0	0	1060	49

Table III: Location and distribution of new cerebral microbleeds after cardiac surgery. Total number of cerebral microbleeds in each location and the total number of CMBs in each location per sample.

**Brain region**	**No. of new CMBs**	**No. of patients with new CMBs in this region (*n*)**	**Percentage of total (%)**
Frontal lobe	136	40	46.1
Parietal lobe	45	27	15.3
Cerebellum	38	19	12.8
Occipital lobe	35	24	11.9
Temporal lobe	23	16	7.8
Hippocampal	4	4	1.4
Putamen/globus pallidus	4	3	1.4
Internal capsule	2	2	0.7
Thalamus	3	2	1
Midbrain	3	3	1
Pons	1	1	0.3
Medulla	1	1	0.3
Total	295	57	100
