# Supplementary material for: Perioperative Cerebral Microbleeds After Adult Cardiac Surgery
Source: Stroke. 2018 Dec 21;50(2):336–43. doi: 10.1161/STROKEAHA.118.023355 (PMC6354910; doi:10.1161/STROKEAHA.118.023355)
Supplement: Supplementary file 2 [file str-50-336-s002.pdf]

American Heart Association | American Stroke Association

Acknowledgment Permission Form

Journal Stroke
Manuscript Number STROKE/2018/023355
First Author Nikil Patel
Title of Work Perioperative Cerebral Microbleeds after Adult Cardiac Surgery

Authors must provide written permission/approval from all individuals mentioned by name in the Acknowledgments section of a submitted manuscript. By signing this form, any and all acknowledged persons therefore state that they have read and approved the mention of their names in the Acknowledgment section of the aforementioned paper.

Name		Signature	Date
Name (1)	Morgan Lillie	M. Lillie	9/10/18
Name (2)			
Name (3)			
Name (4)			
Name (5)			
Name (6)			
Name (7)			
Name (8)			
Name (9)			
Name (10)			
Name (11)			
Name (12)			
Name (13)			
Name (14)			
Name (15)			
Name (16)			
Name (17)			
Name (18)			
Name (19)			
Name (20)			
